# Poor Law Topics

**Published:** 1906-02-10

**Authors:** 


### POOR LAW TOPICS.

### GUARDIANS' CONTRIBUTION TO VOLUNTARY CHARITIES.

It is customary in many districts for the Guardians of the Poor to give contributions to the local charities. The custom is a graceful one, and indicates that those who administer the Poor-law appreciate what is the undoubted fact, that voluntary charities relieve them of much work which would otherwise fall upon the rates. It is perhaps a question if they have the right to give these contributions, but the privilege has never been interfered with. As we have often pointed out, the cases treated by voluntary hospitals and in the Poor-law infirmaries practically are drawn from the same class, and the Guardians' donations may be regarded as a recognition of the assistance which the voluntary institutions give. But a new point of view was recently brought up at Loughborough in connection with a proposal that the Guardians should subscribe three guineas to the Society for the Prevention of Cruelty to Children. After considerable discussion the motion to give the subscription was lost by fifteen votes to five, chiefly on the ground that if public money were voted to the Society its voluntary contributions might diminish. This is a point of view which must be considered. As a matter of fact, very few rate-payers know that these contributions are given; and if they did know, it is unlikely that they would greatly object. But if they knew they might reasonably say, when asked to give privately, that they were already contributing through the poor-rate; and the hard-fisted would probably make that an excuse for refusing to give anything directly. Moreover, there is always the risk—and even where there is no risk there may be a suspicion—that certain charities in which a prominent or energetic Guardian is interested may get more than their share, while others equally deserving are left out. It is indeed a question with several pros and cons, and if it is found that donations by the Guardians are at all likely to limit voluntary gifts, wisdom will suggest that the former be given up. For if voluntary charity dries up, all these charities with their enormous burden of maintenance charges and administrative work will fall upon the rate-payers, on the poorer of whom it will press heavily. The great beauty of voluntary charities lies in the fact that the richer classes of the community maintain them for the benefit of their poorer brethren. If once they are allowed to become dependent on rate or State support it is the poorer rate-payers who will suffer.

A hospital known as the Piletierre Almshouse, belonging to the Little Sisters of the Poor at Rennes, was destroyed by fire on Sunday morning, a number of the inmates being burned to death. The fire, according to accounts from Paris, broke out in the linen closet, and smouldered for hours. Several of the women who were rescued from the flames subsequently succumbed to shock.

Messrs. Bovril, Limited, have recently published a charming photogravure entitled "The Home of the Swans;" (A scene in the Duke of Norfolk's park at Arundel) from the original oil painting by Fred Morgan. This is one of the best of the many excellent gravures produced by the Bovril Company, and can be obtained by collecting the coupons that are given away with each bottle of Bovril.

